# ‘My Advocacy is Not About Me, My Advocacy is About Canadians’: A Qualitative Study of how Caregivers and Patients Influence Regulation of Medical Assistance in Dying in Canada

**DOI:** 10.1093/medlaw/fwae012

**Published:** 2024-04-16

**Authors:** Ruthie Jeanneret, Eliana Close, Jocelyn Downie, Lindy Willmott, Ben P White

**Affiliations:** Faculty of Business and Law, School of Law, Australian Centre for Health Law Research, Queensland University of Technology, Brisbane, Australia; Faculty of Business and Law, School of Law, Australian Centre for Health Law Research, Queensland University of Technology, Brisbane, Australia; Faculties of Law and Medicine, Dalhousie Health Justice Institute, Dalhousie University, Halifax, Canada; Faculty of Business and Law, School of Law, Australian Centre for Health Law Research, Queensland University of Technology, Brisbane, Australia; Faculty of Business and Law, School of Law, Australian Centre for Health Law Research, Queensland University of Technology, Brisbane, Australia

**Keywords:** Assisted dying, Caregivers, Medical assistance in dying, Patient experience, Regulation, Regulatory actors

## Abstract

Medical assistance in dying (MAiD) was legalised federally in Canada after the Supreme Court decision in *Carter v Canada (Attorney General)* [2015] 1 SCR 331. The federal legislative framework for MAiD was established via Bill C-14 in 2016. Caregivers and patients were central to *Carter* and subsequent litigation and advocacy, which resulted in amendments to the law via Bill C-7 in 2021. Research has primarily focused on the impacts of regulation on caregivers and patients. This qualitative study investigates how caregivers and patients influence law reform and the operation of MAiD practice in Canada (ie, behave as ‘regulatory actors’), using Black’s definition of regulation. We found that caregivers and patients performed sustained, focused, and intentional actions that influenced law reform and the operation of MAiD in practice. Caregivers and patients are not passive objects of Canadian MAiD regulation, and their role in influencing regulation (eg, law reform and MAiD practice) should be supported where this is desired by the person. However, recognising the burdens of engaging in regulatory action to address barriers to accessing MAiD or to quality care, and MAiD system gaps, other regulatory actors (eg, governments) should minimise this burden, particularly where a person engages in regulatory action reluctantly.

## I. INTRODUCTION

### A. Overview

This article focuses on patients’ and caregivers’ roles in influencing medical assistance in dying (MAiD) regulation in Canada.^1^ MAiD is known by other terms around the world, including euthanasia, physician-assisted dying, and voluntary assisted dying. MAiD was legalised in Canada after a 2015 Supreme Court of Canada (SCC) decision found the absolute prohibition on physician-assisted dying was unconstitutional.^2^ The subsequent amendment of the Canadian *Criminal Code*^3^ (‘*Criminal Code*’) in June 2016 via Bill C-14 established the MAiD legal framework.^4^ MAiD is defined in section 241.1 of the *Criminal Code* as the administering of a substance to a person by a medical or nurse practitioner, at the person’s request, that causes their death (or prescribing or providing such a substance to a person so that they may self-administer the substance to cause their own death). As of December 31 2022, more than 44 958 people had accessed MAiD in Canada.^5^ Canada is one of a growing number of jurisdictions permitting MAiD.^6^

A central rationale for MAiD is respecting autonomy,^7^ and caregivers are integral in supporting patients to make choices about MAiD.^8^ Despite this, caregivers and patients are often conceived of as passive objects (or ‘regulatees’)^9^ of the MAiD system rather than as having an active role in influencing MAiD laws and how MAiD systems operate. For example, existing literature focuses primarily on the impact of laws, policies, and system design *on* patients’ and caregivers’ experiences,^10^ not their role in influencing their own experiences as well as altering laws, policies, and system design more broadly. No scholarship investigates whether actions of caregivers and patients may constitute regulation (broadly construed) in the Canadian context, though a recent article by Rivest and others has called for further research on involving patients as partners in medical education about MAiD and palliative care.^11^ This absence in literature is problematic given recognition that patient and family caregiver involvement in influencing healthcare may result in better quality healthcare both for individuals and systems.^12^

This article challenges passive conceptions of patients’ and caregivers’ roles as being only objects of MAiD regulation. Using a qualitative, empirical method, we investigate how actions of patients seeking MAiD, and caregivers, may constitute ‘regulation’ of MAiD in Canada. We seek to answer the question: in what ways, if any, do caregivers and patients behave as regulatory actors in Canadian MAiD systems? As will be elucidated further throughout this article, ‘regulation’ is a term that has been defined in many ways in regulatory scholarship. While it has traditionally been conceptualised as law and formal rules, in healthcare (and other) contexts regulation is increasingly understood to be decentred and polycentric, with a much broader range of individuals and tools that influence behaviour.^13^ This study adopts prominent regulatory scholar Julia Black’s definition of ‘regulation’ as sustained, focused, and intentional action to alter behaviour to achieve a particular outcome or outcomes.^14^ Therefore a person who undertakes sustained, focused, and intentional action to alter behaviour is a ‘regulatory actor’.

Based on our empirical findings, we argue (i) caregivers and patients behave as ‘regulatory actors’ in Canadian MAiD systems in a plethora of ways. This means (ii) caregivers and patients should be recognised as actors with voice and agency who can alter Canadian MAiD regulation and are (iii) integral to MAiD law reform and the operation of Canada’s MAiD system in practice. Therefore, (iv) other regulatory actors within Canadian MAiD systems (such as governments) should be responsive to their actions, and ensure the burden of having to take regulatory action to address barriers to access, barriers to quality care, or system gaps does not fall unduly on caregivers and patients, particularly those who act ‘reluctantly’.^15^

Section I(B) of this introduction contextualises patients’ and caregivers’ roles in Canadian MAiD law reform to date. We then consider the literature that defines ‘regulation’ in more detail and what it means to be a ‘regulatory actor’. In Section II, we explain our methodology and in Section III we outline results, including cataloguing the ways caregivers and patients behave as regulatory actors. We then discuss implications of our finding that some caregivers and patients behave as regulatory actors who impact MAiD law reform and the operation of MAiD in practice in Section IV. These implications include that while some may engage in this role willingly and find it meaningful, caregivers and patients should not be required to take action to overcome barriers to access, barriers to quality care, or to address MAiD system gaps,^16^ and thus other regulatory actors within the system (such as governments) should act to minimise this burden.

### B. Canadian MAiD law

The first litigation to challenge the absolute prohibition of MAiD was initiated by Sue Rodriguez, who had amyotrophic lateral sclerosis (ALS).^17^ Rodriguez’s challenge was based on sections 7 and 15 of the *Canadian Charter of Rights and Freedoms (‘Charter’)*; the right not to be deprived of life, liberty, and security of the person except in accordance with the principles of fundamental justice (section 7), and the right to equality (section 15).^18^ While unsuccessful in *Rodriguez* in 1993, the section 7 argument was accepted by the SCC in *Carter* some 20 years later.^19^*Carter* was litigated by multiple plaintiffs including Gloria Taylor, who had ALS,^20^ and the family of Kay Carter, who had spinal stenosis and accessed an assisted death in Switzerland.^21^ The SCC in *Carter* found the blanket prohibition on MAiD unconstitutional: it unjustifiably infringed section 7 by prohibiting MAiD for persons with grievous and irremediable medical conditions causing enduring and intolerable suffering.^22^

After *Carter*, the *Criminal Code* was amended to allow MAiD by Bill C-14, with effect from June 2016.^23^ Bill C-14 introduced eligibility criteria and procedural safeguards for MAiD (Table 1).^24^ It restricted access to MAiD to individuals whose natural death was ‘reasonably foreseeable’, an aspect that was criticised by some academics and others who argued this was vague, overly narrow, and contrary to *Carter*.^25^ Patients were instrumental in challenging the ‘reasonably foreseeable’ natural death requirement.^26^ One *Charter* challenge to this requirement was initiated by Julia Lamb,^27^ who has spinal muscular atrophy.^28^ The most influential *Charter* challenge, though, was *Truchon c. Procureur général du Canada* (‘*Truchon’*),^29^ litigated by two patients with disabilities whose natural death was not reasonably foreseeable: Jean Truchon who had spastic cerebral-palsy with triparesis, and Nicole Gladu who had post-polio syndrome.^30^ Justice Christine Baudouin of the Quebec Superior Court declared the reasonably foreseeable requirement in the *Criminal Code* (and the end-of-life requirement in the Quebec legislation) unconstitutional.^31^ Responding to *Truchon*, Bill C-7 removed the natural death has become reasonably foreseeable eligibility criterion (it remains the characteristic determining which procedural safeguards apply), with effect from March 2021.^32^ This means that individuals whose natural death is not reasonably foreseeable are now able to apply for and access MAiD under the ‘Track 2’ procedural safeguards (Table 1). For individuals whose natural deaths are reasonably foreseeable, the ‘Track 1’ safeguards apply (Table 1).

**Table 1.** Key aspects of MAiD law

	**Bill C-14^a^** *(From June 17 2016)*	**Bill C-7 Track 1 (natural death reasonably foreseeable)^b^** *(From March 17 2021)*	**Bill C-7 Track 2 (natural death not reasonably foreseeable)^b^** *(From March 17 2021)*
***Eligibility criteria***			
Aged 18 years or over and capable of making decisions with respect to their health	**✓** section 241.2(1)(b)	**✓** section 241.2(1)(b)	**✓** section 241.2(1)(b)
Makes a voluntary request, free from external pressure/influence	**✓** section 241.2(1)(d)	**✓** section 241.2(1)(d)	**✓** section 241.2(1)(d)
Provides informed consent to receive MAiD after having been informed of the means that are available to relieve their suffering, including palliative care	**✓** section 241.2(1)(e)	**✓** section 241.2(1)(e)	**✓** section 241.2(1)(e)
Are eligible for health services funded by a Canadian government (or eligible, but for a waiting period)	**✓** section 241.2(1)(a)	**✓** section 241.2(1)(a)	**✓** section 241.2(1)(a)
Have a grievous and irremediable medical condition, ie:	**✓** section 241.2(1)(c)	**✓** section 241.2(1)(c)	**✓** section 241.2(1)(c)
(a) Serious and incurable illness, disease, or disability	**✓** section 241.2(2)(a)	**✓** section 241.2(2)(a) Excludes mental illness—section 241.2(2.1)	**✓** section 241.2(2)(a) Excludes mental illness—section 241.2(2.1)
(b) Advanced state of irreversible decline in capability	**✓** section 241.2(2)(b)	**✓** section 241.2(2)(b)	**✓** section 241.2(2)(b)
(c) Illness or state of decline causes enduring intolerable suffering (physical or psychological) that cannot be relieved under conditions deemed acceptable by the person	**✓** section 241.2(2)(c)	**✓** section 241.2(2)(c)	**✓** section 241.2(2)(c)
(d) Natural death has become reasonably foreseeable	**✓** section 241.2(2)(d)—now repealed	**✗** Reasonably foreseeable natural death is no longer part of the eligibility criteria but is still relevant in assessing whether the Track 1 or Track 2 safeguards apply.	**✗** Reasonably foreseeable natural death is no longer part of the eligibility criteria but is still relevant in assessing whether the Track 1 or Track 2 safeguards apply.
***Procedural safeguards***			
First eligibility assessment by MAiD assessor/provider (either a medical or nurse practitioner)	**✓** section 241.2(3)(a)	**✓** section 241.2(3)(a)	**✓** section 241.2(3.1)(a)
Second eligibility assessment by either a medical or nurse practitioner (who the first assessor/provider is satisfied is independent), who provides a written opinion confirming the person meets all of the eligibility criteria	**✓** section 241.2(3)(e) and (f)	**✓** section 241.2(3)(e) and (f)	**✓** section 241.2(3.1)(e) and (f)
Patient makes a written request which is independently witnessed, signed and dated	**✓** Two witnesses section 241.2(3)(b) and (c)—now amended	**✓** One witness section 241.2(3)(b) and (c)—as amended	**✓** One witness section 241.2(3.1)(b) and (c)—as amended
Both assessors must inform the patient they can withdraw their request at any time	**✓** section 241.2(3)(d)	**✓** section 241.2(3)(d)	**✓** section 241.2(3.1)(d)
If the person has difficulty communicating, take all necessary measures to provide a reliable means by which the person may understand the information provided to them and communicate their decision	**✓** section 241.2(3)(i)—now amended (this requirement is now included in s 241.2(3)(g))	**✓** section 241.2(3)(g)—as amended	**✓** section 241.2(3.1)(j)
Waiting period	**✓** 10 clear days between written request and provision. Period could be shortened if death or loss of capacity imminent. section 241.2(3)(g)—now repealed	**✗**	**✓** 90 clear days between first assessment and provision. Period can be shortened if loss of capacity to consent to MAiD is imminent. section 241.2(3.1)(i)
Immediately before providing MAiD, give the person an opportunity to withdraw their request and ensure that the person gives express consent to receive MAiD (ie, final consent requirement)	**✓** section 241.2(3)(h)	**✓** section 241.2(3)(h) **Provider-administered MAiD:** Final consent not needed if the criteria is met for a waiver of final consent (a written arrangement entered into prior to loss of capacity). The substance can be administered on the terms of the arrangement, unless the person demonstrates, by words/sounds/gestures refusal to have the substance administered or resists administration. section 241.2(3.2), (3.3), and (3.4) **Self-administered MAiD:** Final consent not needed if advance consent for provider-administered MAiD after failed self-administration (ie, a written agreement entered into prior to loss of capacity) exists. section 241.2(3.5)	**✓** sections 241.2(3.1)(k) **Provider-administered MAiD:** Final consent must be provided (a waiver of final consent is unavailable). **Self-administered MAiD:** Final consent not needed if advance consent for provider-administered MAiD after failed self-administration (ie, a written arrangement entered into prior to loss of capacity) exists. section 241.2(3.5)
If neither of the assessors has expertise in the condition causing the person’s suffering, consult a medical or nurse practitioner who has the requisite expertise	**✗**	**✗**	**✓** section 241.2(3.1)(e.1)
Informed, by the first and second assessors/providers, of means available to relieve suffering, including counselling services, mental health and disability support services, community services and palliative care, and offered consultations with relevant professionals who provide those services or care	**✗**	**✗**	**✓** section 241.2(3.1)(g)
Agreement between the first and second assessor/provider that the person has given serious consideration to means available to relieve suffering	**✗**	**✗**	**✓** section 241.2(3.1)(h)

a Bill C-14, An Act to Amend the Criminal Code and to Make Related Amendments to Other Acts (Medical Assistance in Dying), SC 2016, c 3.

b Bill C-7, *An Act to Amend the Criminal Code (Medical Assistance in Dying)*, SC 2021, c 2.

Bill C-7 made other changes to the law in response to advocacy efforts,^33^ including allowing an exception to the requirement to provide final consent immediately prior to MAiD provision in some cases (Table 1). The latter amendment is often referred to as ‘Audrey’s Amendment’.^34^ This reflects the powerful advocacy of Audrey Parker (supported by friends), including a poignant video filmed just prior to her death, advocating for the final consent requirement to be removed.^35^ Audrey had Stage IV breast cancer and died through MAiD earlier than she wanted for fear of losing the capacity to provide final consent.^36^ Many other caregivers and patients have advocated for reform too, demonstrated by Dying With Dignity Canada’s (DWDC) blog page,^37^ and testimony before parliamentary committees, including the Special Joint Committee hearings on MAiD.^38^ Caregivers and patients have evidently been instrumental in Canadian MAiD law reform both in terms of litigation^39^ and wider advocacy: their actions directly contributed to MAiD legalisation through Bill C-14, and they also drove changes implemented by Bill C-7.^40^

### C. Other sources of Canadian MAiD regulation

The legal framework in the *Criminal Code* is the foundation of Canadian MAiD regulation. ‘Regulation’ is sometimes traditionally understood to refer to the law made by the state,^41^ and in the Canadian healthcare context is often used to refer to the activities of formal bodies (ie, ‘formal regulators’), such as medical or nursing colleges setting and enforcing standards for behaviour.^42^ But regulation is increasingly recognised as being broader than this, encompassing a range of sources and actors that influence behaviour, beyond formal sources.^43^ We use Black’s broad definition of regulation:

…the sustained and focused attempt to alter the behaviour of others according to defined standards or purposes with the intention of producing a broadly identified outcome or outcomes, which may involve mechanisms of standard-setting, information-gathering and behaviour-modification.^44^

Black’s definition has three elements: (i) sustained and focused action; (ii) directed towards altering others’ behaviour; and (iii) intention to produce a particular outcome.^45^ Black does not limit regulation based on who is performing the action, whether the action successfully achieves an outcome, or whether a person can enforce that outcome. Instead, this definition focuses on *what* people are doing and *why*. Black’s definition of regulation is described as a decentred or polycentric account of regulation because it recognises that the state is not the sole source of regulation, and that many different individuals and tools impact regulation.^46^

Decentred and polycentric definitions of regulation are apposite for healthcare (eg, MAiD) where no single entity has the power and knowledge required to regulate effectively.^47^ There is clear evidence of non-state actors influencing regulation of MAiD in Canada since its legalisation. For example, regulatory gaps resulting from the federal-provincial MAiD regulatory patchwork (ie, MAiD is regulated by federal criminal law, but also supplementary provincial and territorial health legislation, policy, and other sources) have been filled by non-government individuals and entities. These include the Canadian Association of MAiD Assessors and Providers (CAMAP) (the national professional body, which grew from a need ‘to establish training resources, to create medical standards, and to encourage the standardisation of care’^48^ and has developed a national clinical MAiD curriculum)^49^; DWDC (the national peak body representing individual interests relating to MAiD and other end-of-life choices) which plays an important role including by distributing knowledge about MAiD,^50^ advocating for improved access, and organising witnesses^51^; and individual health institutions and practitioners.^52^ These organisations, while not formal ‘regulators’ enforcing behaviour through law or formal rules, are arguably ‘regulatory actors’ who take sustained and focused action, directed towards altering others’ behaviour, with the intention of producing particular outcomes.

This raises the central question relevant here: can caregivers and patients be regulatory actors too? The role of caregivers and patients in regulating healthcare has scarcely been investigated,^53^ and is contested.^54^ Healy describes that historically patients have, paradoxically, been the ‘missing person’ in discussions about healthcare regulation.^55^ Yet, there is clear evidence of caregivers and patients (eg, Kay Carter, Gloria Taylor, Jean Truchon, Nicole Gladu, Julia Lamb, and Audrey Parker) impacting MAiD law reform in Canada, and therefore influencing regulation. A cognate study investigating this question in an Australian state’s assisted dying system also found that some caregivers and patients acted as regulatory actors.^56^ This article investigates the ways in which caregivers and patients may be seen to behave as regulatory actors in Canada, adopting Black’s definition of regulation.

## II. METHODOLOGY

### A. Study design

This study is nested within an international study examining MAiD regulation in Australia, Canada, and Belgium.^57^ We adopt a critical realist approach.^58^ This article reports experiences of caregivers and patients in influencing Canadian MAiD regulation, drawing on data collected through qualitative interviews with a patient and caregivers.

Ethics approval was obtained from the Queensland University of Technology University Human Research Ethics Committee (#2000000270) and the Dalhousie University Research Ethics Board (#2020-5313 and #2021-5688). This study is reported according to COREQ guidelines.^59^

### B. Eligibility and recruitment

Patients were eligible to participate if they had requested MAiD and begun the assessment process, irrespective of the outcome or whether the process was complete. Caregivers were eligible if they had supported a patient through the MAiD process. For feasibility, participants were recruited from three provinces, selected to provide geographic and regulatory diversity (MAiD implementation varies by province and territory): British Columbia, Ontario, and Nova Scotia.^60^

Initially, we relied on convenience sampling to recruit participants who contacted us in response to recruitment material shared via DWDC mailing lists, our website, and Twitter.^61^ We also used purposive sampling to ensure diversity,^62^ including DWDC contacting individuals with experiences we were seeking (eg, non-cancer diagnoses), and the research team contacting individuals with whom they had pre-existing connections. We also used snowball sampling.^63^ Recruitment ceased when the research team were satisfied there was sufficient ‘information power’.^64^

### C. Data collection

Data were collected via qualitative, semi-structured interviews. The research team developed interview guides for each cohort (caregivers and patients). Interview guides were similar but contained some different questions, reflecting the different nature of experiences (Supplementary Material). Interviews covered issues relevant to the broader international study, such as about the MAiD request and assessment process. These questions were relevant in answering the question investigated in this article because they provided an overarching picture of the person’s MAiD experience. However, prompts were also used to elicit information specifically about potential regulatory action. For example, participants were asked: *‘Were there any roadblocks? Disagreements? Difficulties accessing MAiD? What was done to get past this? Prompt (if disagreement or barrier): Explore if they initiated complaint process, formal or informal, and how this was initiated.’* Because of our semi-structured approach to interviews, we asked additional questions to clarify and probe areas of interest or complexity raised by participants.

RJ led all interviews, with JD participating in two early interviews (including the patient interview) to test and refine interview guides and ensure appropriateness within the Canadian context. EC participated in a further 11 interviews as part of RJ’s research training, to iteratively refine the interview guides, and inform other aspects of the broader Canadian study. One of these 11 interviews was jointly conducted by RJ and EC because the participant had a dual role as a caregiver and healthcare professional, i.e., they had supported a family member to access MAiD, and separate to this experience, they are a MAiD assessor/provider (relevant to the broader study). Interviews were conducted via Zoom between October 9 2021 and August 5 2022. All participants provided informed consent. All caregiver interviews occurred after the patient who they supported died, so consent from patients was not sought. RJ debriefed after each interview with other members of the research team, and RJ and EC maintained a reflexive journal.

### D. Data analysis

Interviews were recorded and professionally transcribed verbatim. Transcripts were sent to participants (member checking).^65^ Six participants provided supplementary information by email, which was included in the analysis. All transcripts and supplementary information were deidentified and uploaded to NVivo (QSR International, release 1.6.1) for analysis using codebook template analysis (some themes were developed deductively, with inductive and iterative theme refinement).^66^

Our analysis drew on an analytical framework developed by RJ, EC, LW, and BW in a cognate study from Victoria, Australia.^67^ RJ initially deductively coded data into four overarching *a priori* themes: actions, motivations, impacts, and mediating factors. Once these data were identified, RJ inductively coded the data again line by line. A second author (EC) reviewed the subset of data to enrich analysis and iteratively refine the thematic framework, including exploring possible diverse interpretations of the data.^68^ The four themes reported here mirror the Victorian study. Both surface-level (semantic) and underlying (latent) themes are reported.

## III. RESULTS

### A. Overview of results

Thirty-two interviews were conducted with 34 participants between October 2021 and August 2022 (1 patient, 33 caregivers; Table 2) about 33 patient experiences of MAiD (Table 3). Two interviews involved two caregivers (eg, a son and daughter-in-law), and one participant spoke about two patients. All caregivers were supportive of MAiD in principle and of the patient’s choice to access MAiD (expressions of supportiveness were sometimes explicit and sometimes implicit). The sole patient interview was conducted with a patient who was in the process of being assessed for MAiD. Interviews ranged between 55 and 203 min, with a median of 83.5 min.

**Table 2.** Characteristics of interview participants (*n* = 34)

Characteristics	Number (*n* = 34)
**Age (years), mean 58.6**	
20–29	1
30–39	2
40–49	6
50–59	8
60–69	8
70–79	7
80–89	2
**Gender**	
Female	25
Male	9
**Relationship to patient** ^a^	
Child (including stepchild, child in-law)	17
Spouse (including de facto partner)	12
Parent	2
Close friend	2
Niece/nephew	1
Self	1

a One participant reported two cases, so this category is calculated using the total number of relationships (*n* = 35).

**Table 3.** Characteristics of patients described by interview participants (*n* = 33)

Characteristics	Number (*n* = 33)
**Age (years), mean 70.8**	
30–39	2
40–49	2
50–59	1
60–69	6
70–79	12
80–89	9
90–99	1
**Gender**	
Female	18
Male	15
**Illness type**	
Cancer	20
Neurological	6
Other (eg, COPD, heart disease)	7
**Province**	
Ontario	14
British Columbia	11
Nova Scotia	6
Other^a^	2
^a^The participants who reported on patient cases relating to other provinces were either located in one of the target provinces, or also reported on a patient case that occurred in one of the target provinces, hence inclusion in our sample.	
**Location of death**	
Home	17
Hospital^a^	8
Long-term care facility^b^	3
Abortion clinic^c^	1
Hospice	1
Nursing home	1
Assisted living facility	1
Not applicable	1
^a^Two patients died in hospital but not from a MAiD death. ^b^One patient died in the long-term care facility but not from a MAiD death. ^c^This patient was transferred from a long-term care facility on the day of MAiD administration.	
**Timing of death**	
2016	1
2017	5
2018	10
2019	3
2020	5
2021	8
Currently seeking MAiD	1
**Patient’s education level**	
Postgraduate university	12
Undergraduate university	6
University diploma	1
Some university (not completed)	3
Community college	3
High school	5
Some high school	3

**Figure 1.**
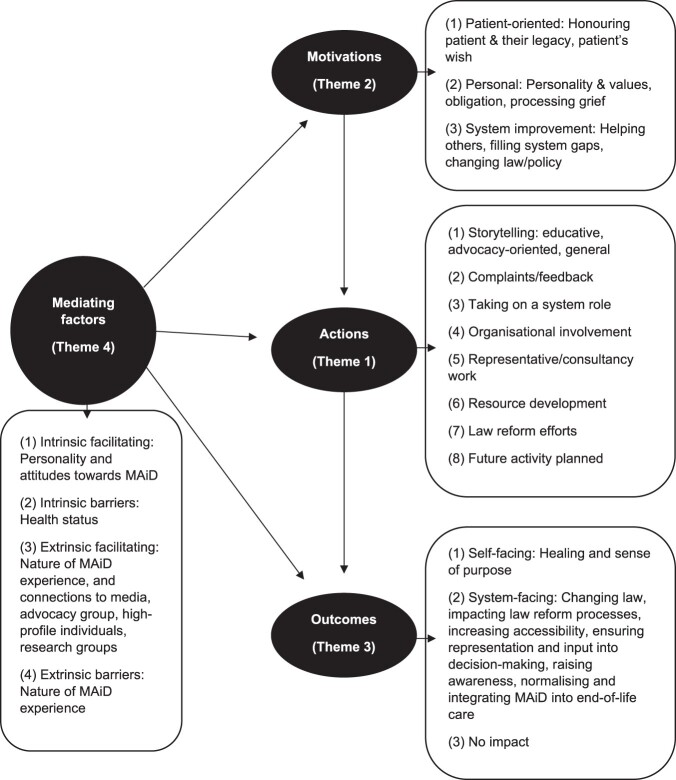
The relationship between the four themes, eg, how mediating factors (Theme 4) relate to actions (Theme 1), motivations (Theme 2), and outcomes (Theme 3). It also lists the sub-themes within each theme.

Using the analytical framework developed in the Victorian cognate study,^69^ we developed four themes: (i) potentially regulatory actions by caregivers and patients; (ii) motivations for actions; (iii) outcomes of actions; and (iv) mediating factors, which impact on actions, motivations, and outcomes. These themes overlap (Fig. 1). Reflecting this study’s focus on regulation (broadly defined), this analysis is focused on patient and family caregiver actions to influence the MAiD system, rather than on efforts to influence the individual healthcare relationship.

### B. Theme 1: potentially regulatory actions

Participants (and the patients they described) performed potentially regulatory actions, including: (i) storytelling; (ii) complaints and feedback; (iii) taking on a system role; (iv) organisational involvement; (v) representative and consultancy roles; (vi) resource development; (vii) law reform efforts; and (viii) future activity planned.

#### 1. Storytelling

Caregivers publicly shared their story of supporting a patient through various media such as television series and interviews, documentaries, podcasts, webinars, videos, presentations, workshops, live storytelling, eulogies, newspaper and magazine articles, blog posts, obituaries, and books:

I give testimonials of [patient]’s story… (2108)

The sole patient participant also shared their story of their illness and desire to access MAiD:

…I just told them…this is what I've been going through, and I would value this… (2103)

Often storytelling had an educative function. For example, one caregiver attended a hospital ‘before-rounds’ session to inform staff about their family member’s experience. Sometimes, storytelling was advocacy oriented. For example, one participant used their family member’s experience ‘as a point of advocacy’ to increase MAiD knowledge; one ‘sponsored’ a MAiD workshop and invited a local parliamentarian; and one met with a university medical school dean, to get ‘more doctors trained in end of life’.

These actions overlap with Theme 2 as motivations are inextricably linked to the type of storytelling (educative, advocacy-oriented, or general).

#### 2. Complaints and feedback

Participants provided feedback to politicians, health professionals, institutions, and funeral homes about suboptimal aspects of MAiD experiences (eg, delays collecting the body post-death, communication around the administration process). One participant, whose family member faced a forced transfer due to an institutional objection to MAiD by a faith-based institution:

…worked with the BCCLA [British Columbia Civil Liberties Association]…The rabbi of the synagogue wrote a letter. I wrote a letter. [The patient] wrote a letter. (2128)

One participant felt their feedback was being ignored, prompting them to create an online forum to discuss feedback and share information.

Participants also saw participation in this study as providing feedback to contribute to shaping research on MAiD and improving practice.

No one made formal complaints, such as to regulatory colleges, or engaged in litigation, though several participants reported litigation was considered:

…it was clear…we could go to court…And she [patient] looked at me and she said, ‘I'm too tired,’ and there were tears in her eyes. (2128)

#### 3. Taking on a system role

Some participants had taken on roles within the MAiD system, including as volunteer witnesses with DWDC, in senior roles within DWDC, and with MAiD peer support organisations including DWDC, Bridge C-14, and Bridge4You. One participant (a health professional) became an MAiD provider partly because of delays their family member experienced in arranging MAiD provision:

…seeing how it affected my [family member] having to wait…did give me pressure… (2126)

Two other participants (end-of-life doulas) incorporated their lived experience into their work:

…it’s just amplified the work and the skill that I'm already doing. (2106)

#### 4. Organisational involvement

One participant attempted to alter an institution’s position on forced transfers by becoming a shareholder in the institution objecting to providing MAiD onsite.

#### 5. Representative and consultancy work

Participants undertook representative and consultancy roles, to represent lived experience as a caregiver of a person who accessed MAiD, in organisations including CAMAP; DWDC’s First-Person Advocates’ Initiatives Council (for individuals who have supported a person through MAiD); and provincial, national, and international governments.

Participants also attended academic conferences and were involved in research grants as consumer representatives. One participant highlighted the importance of lived experience in academic circles:

We live it. Instead of telling us what you think we need, why don't you ask us not only what we need but what we want? (2110)

#### 6. Resource development

Related to representative and consultancy roles was resource development. For example, two participants advised on a new clinician MAiD curriculum.

Another participant worked with an advocacy organisation to develop resources for people preparing to access MAiD, or supporting someone through the process:

I’m helping…put together a to-do list and a checklist of things that we've experienced… (2105)

On their own initiative, one participant wrote two books on talking to children about MAiD and grief:

…that need for something…that's child friendly, just came through so loud and clear…I just felt moved to create these story books. (2106)

#### 7. Law reform efforts

Several caregivers and patients provided information, wrote letters, wrote briefs to members of parliamentary committees, or appeared in parliamentary proceedings as witnesses. Caregivers and patients were eager to be politically involved. One patient sent an email to every member of Parliament at a federal, provincial, and municipal level.

Some participants attended political events or wrote to politicians to provide feedback:

…I showed up [at a political event] and I chatted with [Minister]…I was very much against her restrictions in Bill C-14… (2103)

One participant described concerted efforts (including media efforts and direct contact with legislators) by a patient, supported by caregivers and DWDC, to change the final consent requirement. The patient knew law reform efforts would not succeed in time for them to benefit, so asked the participant (and others) to continue advocating after their death:

…‘This is what you’ve got to do… you’re going to fight for this.’ (2121)

No participants reported initiating litigation directed at law reform.

#### 8. *Future activity planned*

Some participants planned to engage in future action but were grappling with how and if they wanted to be involved. Some encountered barriers to involvement such as having ‘to wait a year before you can be available to consult [in a peer support role]’ (2105).

### C. Theme 2: motivations for undertaking actions

Participants reported various motivations for undertaking actions in Theme 1, divided into patient-oriented, personal, and system improvement motivations. Often, motivations were multifaceted and spanned across multiple categories.

#### 1. Patient-oriented motivations

The primary patient-oriented motivation expressed^70^ by caregivers was honouring the patient:

I feel like it’s [storytelling]…honouring my dad. (2101)

This motivation was expressed in various ways. One participant commented ‘people love to be remembered and people love to remember’ (2121), with another explaining:

…that’s my way of keeping [patient] alive, by being able to share her story… (2108)

Some family caregiver participants upheld the patient’s legacy in a specific way that honoured the patient’s values:

[Advocacy opportunities have been] amazing because it’s exactly what [patient] would have wanted, having been a fighter and a rabble rouser her entire life… (2128)

Other family caregiver participants said patients’ ‘dying request’ or expectation was they try to improve the MAiD system; a task they felt privileged to undertake:

[Patient]…took my hands and said ‘Please don’t let me have died for nothing. Make my death have meaning. Tell our story.’ (2110)

#### 2. Personal motivations

Personal motivations included intrinsic personality and values, feelings of obligation, and (for caregivers) processing grief.

Regarding intrinsic personality and values, many participants tried to alter the MAiD system because it had become their ‘purpose’ or ‘mission’ or was part of their ‘third act’ (ie, a period of life focused on contribution). One explained activism is ‘just who I am’. Another reflected the MAiD experience had ‘found’ her and, because it had, she took the opportunity to use the experience productively (as she described, by turning ‘lemons into limoncello’).

A second motivation was feeling an obligation to act:

…if I can be the voice for others…then I feel that’s an obligation… (2110)

A third motivation was grief, particularly the ‘very specific kind of grief’ and ‘surreal experience’ associated with MAiD. Some participants’ efforts to influence the system were to process grief with one finding this ‘an extremely useful tool in the grieving process’. It also resulted in connections to organisations (such as DWDC, Bridge C-14, or Bridge4You (now referred to as MAID Family Support Society)) which provided opportunities to amplify their efforts. For example, one participant said:

…if [patient]’s leaving us too soon can in some way help others, I am so open to sharing his story, partly because yes, it’s healing for me… (2107)

Another participant commented that while many advocates are motivated by altruism, there is an element of advocacy about personal healing because, inherently, advocacy to make something better suggests an aspect of a person’s own experience was suboptimal, and advocacy may partially be about reconciling that.

#### 3. System improvement motivations

Most motivations to improve the system (from participants and/or patients they described) fell into three categories: (i) helping and empowering other caregivers and patients, (ii) filling system gaps, and (iii) changing laws and policies. Often this was intertwined with patient-oriented or personal motivations. For example, some actions directed at bringing about system change were also motivated by honouring a patient.

##### a. Helping and empowering other caregivers and patients

A primary system improvement motivation was helping other caregivers and patients to navigate MAiD.^71^ This included spreading awareness, destigmatising, educating, and advocating about MAiD as a lawful option:

I want to be able to take [patient]’s experience and help others get to that same point, as many as I can… (2120)

Participants reported stigma was prevalent after MAiD was legalised, and though things had improved, stigma remains:

…I’m currently working with three patients who are struggling to get assessed…you’re running up against a lot of different prejudices and stigmas even six years down the road… (2110)

Often participants expressed the motivation to help others more generally:

…if we’ve lost someone, we want to help others to go through this process… (2108)

Sometimes, it was more specific and linked to a particular means of helping others:

It was very clear instantly how fortunate she was to have two people who were honoured to sign…that fuelled me…to register as an independent witness… (2113)

…I thought it [storytelling] might help other immigrant families of colour and Muslims. None of these things are mutually exclusive from you being able to have an assisted death… (2102)

##### b. Filling system gaps

Many participants recognised a gap in the MAiD system during their MAiD experience, and they wanted to fix it for others, for example, the participant who wrote books about MAiD-related grief support for children.

Another gap was the ability to speak with a peer, leading to individuals offering their support in peer support roles. One participant shared her experience of supporting a family member to access MAiD in a media interview early in the legalisation of MAiD:

…I thought ‘This would have been so welcome to me when I was at that stage to actually hear from somebody who had been through it… (2114)

##### c. Change laws and policies

Participants were motivated to change laws and policies. This sentiment is encapsulated in an example provided by a caregiver, who supported a patient through MAiD who had publicly advocated for legal change. The participant explained there was a point during this advocacy when the patient realised her motivation shifted from changing the law for her benefit, because it would happen too late, to improving the law for others:

…there was a point where she realised, okay, my advocacy is not about me, my advocacy is about Canadians. (2121)

Another example is a caregiver and patient writing letters to the patient’s long-term care facility to try to change the institutional policy prohibiting MAiD onsite:

…it was about making a path for people to be able to access this safe, comforting, incredibly helpful medical procedure. It wasn’t just about [patient]… (2128)

Another family caregiver with legal expertise helped draft guidelines to promote referrals by objecting health professionals.

### D. Theme 3: outcomes of actions by caregivers and patients

Actions in Theme 1 had: (i) outcomes for the individual taking the action (self-facing), (ii) outcomes for the system more widely (system-facing), and (iii) no impacts.^72^ This theme intersects with Theme 2.

#### 1. Self-facing

The primary self-facing outcomes^73^ were providing a sense of purpose and/or assisting with grief:

The work with Dying With Dignity has been…an extremely useful tool in the grieving process…helping a little bit really feels healing… (2128)

…that advocacy…gave purpose again. (2121)

#### 2. System-facing

System-facing outcomes can broadly be divided into two categories: (i) impacting MAiD law reform, and (ii) influencing the operation of MAiD in practice.

##### a. Impacting MAiD law reform

Actions of caregivers and patients impacted law reform and political processes. The most significant example was a patient directly influencing the final consent requirement for all MAiD cases (ie, an exception is now available in some cases).

Several participants influenced law reform by providing personal accounts to members of parliament, impacting parliamentary debates. For example, one participant worked with advocates and lawyers to develop a document sent to parliamentarians about the prohibitive effect of a judicial pre-authorisation requirement (a requirement which was ultimately not included in law). The participant also submitted a document based on her experience supporting her family member, which was cited in parliament:

I’m…listening to this, like oh my gosh, she’s talking about my [family member]…The vote comes back in, and it’s defeated. (2102)

##### b. Influencing the operation of MAiD in practice

Actions of patients and families influenced the functioning and operation of MAiD in practice. Participants perceived their actions improved accessibility. For example, many participants volunteered as witnesses, with one having done ‘about 25’. Participants in representative or consultative roles helped to improve resources by increasing readability:

…I said ‘If you’re an immigrant…if you’re an Aboriginal person living in a remote area…, can you access that information, do you understand it…?…’ They…rewrote it in accessible language… (2110)

Engagement in representative or consultative roles also meant caregivers and patients had input into decision-making about MAiD practice.

Another outcome was raising MAiD awareness. Sometimes, participants educated healthcare professionals who were unaware MAiD was legal (reported more often by participants with earlier MAiD experiences). One caregiver alerted a doctor to the MAiD coordination team within the hospital. By doing this, participants considered they increased knowledge and awareness among health professionals, which may have impacted their professional practices and their approach to treating other patients seeking MAiD (ie, altered or improved their practice). This also extended to funeral homes, who were provided with feedback. For participants who were health professionals themselves, they described incorporating their lived experience into (thereby improving) their own professional practice.

Not only did participants inform health professionals that MAiD was legal, but they also spread broader MAiD awareness. Outcomes from this were others: (i) thanked them for sharing information and/or (ii) accessed MAiD after becoming aware of the option from stories shared by participants and patients:

…since I told…our story, people come to me for information…I’ve been personally involved in another four, I’ve actually provided information for many more… (2114)

The culmination of outcomes outlined above contributed to normalisation of MAiD and integration into end-of-life care.

#### 3. No impact

Some caregivers and patients indicated their actions did not change MAiD law or its operation in practice. One participant indicated that a challenge to an institutional policy requiring a forced transfer was unsuccessful:

Unfortunately, I don’t think that they’re going to respond to heartfelt pleas or goosebump-inducing writing or even threats of legal action…The only thing that will make that difference is legislative… (2128)

### E. Theme 4: mediating factors

A range of intrinsic and extrinsic factors mediated participants’ and patients’ ability to take actions to alter the MAiD system (Theme 1). These factors also affected a person’s motivations (Theme 2) and whether they could achieve the outcome they sought (Theme 3).

#### 1. Intrinsic mediating factors

One intrinsic mediating factor *facilitating* action was the individual’s personality and attitude towards MAiD. A key aspect was supportiveness of MAiD: all participants were in principle supportive of MAiD and of their family members’ choice to access MAiD, though some experienced reservations or complexities within their experience. This factor overlaps with Theme 2 but is reported here because it affects whether they would undertake actions, and what kind (Theme 1). For example, participants were motivated to volunteer as MAiD witnesses because of their support for MAiD in principle.^74^

Other aspects of personality and attitudes towards MAiD also *facilitated* action: it was in their ‘nature’, or they were just ‘that kind of guy’, with one participant referring to themselves as an ‘activist’. Another said:

I’m as outspoken as my mother …it’s part of the reason that I’ve contacted Dying With Dignity… (2129)

Conversely, an intrinsic mediating factor that was a *barrier* to action was health status. This was exemplified by one participant who described the patient declining to litigate saying ‘I’m too tired’ (due to the progression of her illness on her health).

When a patient was too unwell to act, they engaged others to assist. One participant described the patient engaging their assistance with their advocacy ambitions: the patient advocated for law reform and realised they would not be able to see the results of this advocacy due to illness progression, so said to the participant: ‘This is what you've got to do…you're going to fight for this’ (2121).

#### 2. Extrinsic mediating factors

One extrinsic mediating factor that was both a facilitator and a barrier was the nature of a person’s experience, including whether they experienced roadblocks or gaps (ie, facing a barrier such as institutional objection, or lacking support).

The nature of a person’s MAiD experience sometimes *facilitated* action, and affected the type of action they took (linked to Theme 2). Often, a person’s actions were directed at overcoming similar roadblocks or filling system gaps they experienced. A clear example is participants joining peer support organisations or sharing stories because this would have been ‘welcomed’ during their own experience. Another example is the participant who wrote books on grief because those resources were absent for them. System maturity at the time of the person’s MAiD experience was also relevant, with some commenting they acted because the system was so new.

In some instances, however, a difficult MAiD experience was a *barrier*. For example, one participant was emotionally drained by the difficult MAiD process (caused by the long-term care facility’s stance on MAiD and obstructive behaviour) and felt like they could not engage as their energy was expended supporting the patient’s choice for MAiD:

I really grappled with actually giving some pretty serious feedback…But…you’re emotionally drained…in the end you decide to just let it go. (2112)

A second extrinsic factor *facilitating* action was connections to advocacy groups,^75^ high-profile individuals, media, or research teams.

In relation to advocacy groups, many participants specifically mentioned a relationship with DWDC, or high-profile individuals within DWDC, including one participant previously holding a senior position with DWDC. This connection *facilitated* actions or amplified outcomes because of DWDC’s reach and status as a national organisation.

In contrast, the absence of DWDC in their region prompted one participant to act:

…we're very disconnected from the Ontario headquarters…we have our own little world out here. (2103)

Connections to other high-profile individuals or organisations external to DWDC also *facilitated* actions. For example, one participant said:

…one of our members of Parliament is a personal friend and she wanted to know [patient]'s story and she presented [patient]’s story… (2104)

Other high-profile individuals and organisations mentioned included key legal academics, the British Columbia Civil Liberties Association (BCCLA), CAMAP, Bridge C-14, Bridge4You (now known as MAID Family Support Society), religious leaders, university professors, care coordinators, and other individuals such as MAiD advocates or persons with lived experience.

Media attention also had a significant *facilitating* impact and provided opportunities to undertake action, such as podcasts, television interviews, and newspaper articles. It also amplified the voices of patients and participants by increasing the reach of actions, making it more likely they could achieve their desired outcome (Theme 3):

…the media got involved and the newspaper in [location] devoted the whole front page…everywhere I went people would stop and thank me…for having had the courage to be so public… (2110)

And finally, some participants were involved in research teams and on research grants which *facilitated* action:

…the researchers wanted to include us and our perspective…they really value that… (2113)

## IV. DISCUSSION

### A. Overview of findings

Caregivers and patients engaged in actions, including storytelling, making complaints, providing feedback, and law reform-oriented actions, that can be defined as ‘regulatory’ according to Black.^76^ They were motivated to act by factors including wanting to help others, filling system gaps, or changing laws or policies. Participants perceived their actions (i) impacted Canadian MAiD law reform and (ii) influenced the implementation, delivery and functioning of MAiD in Canada (ie, the operation of MAiD in practice). Sometimes, though, actions had no outcome and participants perceived stronger forms of regulation (eg, laws) were necessary to achieve change.

The range of regulatory actions reported by participants was broad. Regulatory actions were directed towards law reform or to the operation of MAiD in practice. By directed towards ‘law reform’, we mean that some regulatory actions were directed at changing the law to allow MAiD or, post-legalisation, to overcome perceived legal barriers by changing the law (eg, the final consent requirement). Other regulatory actions were not directed at changing the law, but rather at the operation of MAiD in practice within the existing legal framework. This included overcoming barriers to access in practice (eg, volunteering as an independent witness) or filling gaps in regulation (eg, creating resources). While some categories of actions described in Theme 1 were directed at just one of these aims, eg, the action we have described as ‘law reform efforts’ is solely directed at altering law reform, most actions could be directed at either depending on the circumstances. For example, ‘storytelling’ could be educative and aimed at spreading awareness about MAiD to make the option of MAiD more accessible (directed at altering the operation of MAiD in practice), but it could also be advocacy-oriented storytelling directed at drawing attention to a legal barrier, which could only be resolved through law reform. Table 4 highlights the relationship between examples of regulatory actions (Theme 1) undertaken by patients and families, how they link to aspects of the MAiD process, and how these actions relate to either impacting law reform or influencing the operation of MAiD in practice (Theme 3).

**Table 4.** Relationship between regulatory actions, outcomes, and aspects of the MAiD process

Aspect(s) of MAiD process	Examples of regulatory actions (Theme 1)	Examples of system-facing outcome/intended outcome (Theme 3)
***Information about MAiD and beginning the process***		
Information about MAiD	**Storytelling**: Sharing information in TV series, interviews, documentaries, podcasts, webinars, videos, presentations, workshops, live storytelling, eulogies, newspaper articles, magazine stories, blog posts, obituaries, books, personal networks, workplaces	Influencing the operation of MAiD in practice, including by: Raising awareness of MAiD amongst the public Informing health professionals about MAiD Normalising MAiD and integrating it into end-of-life care
	**Storytelling**: Sponsoring a workshop/forum to share information	Influencing the operation of MAiD in practice, including by: Raising awareness of MAiD amongst the public Normalising MAiD and integrating it into end-of-life care
	**Complaints and feedback**: Creating online forum to share information and feedback among individuals with lived experience	Influencing the operation of MAiD in practice, including by: Improving accessibility of information about MAiD
Connecting to a willing provider	**Representative and consultancy work**: Helping to develop referral requirements for objecting clinicians	Influencing the operation of MAiD in practice, including by: Improving accessibility of MAiD
***Request and assessment process***		
Written request	**Taking on a system role**: Volunteering as independent witnesses	Influencing the operation of MAiD in practice, including by: Improving accessibility of MAiD
Eligibility criteria	**Storytelling**: Sharing story to explain why they would value expansion of criteria from their personal experience	Impacting MAiD law reform
	**Law reform efforts**: Advocacy for reform to eligibility criteria (eg, natural death has become reasonably foreseeable)	Impacting MAiD law reform
	**Law reform efforts**: Participating as a witness in parliamentary committees and providing evidence about eligibility criteria issues	Impacting MAiD law reform
	**Law reform efforts**: Briefs/evidence to parliamentary committees relating to eligibility criteria (eg, to allow advance requests)	Impacting MAiD law reform
Safeguards	**Law reform efforts**: Advocacy for removal of safeguards perceived as barriers to access or as unconstitutional (eg, final consent in all MAiD cases requirement)	Impacting MAiD law reform
	**Law reform efforts**: Being a witness in parliamentary committees regarding safeguards and process (e.g., to allow advance requests)	Impacting MAiD law reform
	**Law reform efforts**: Briefs to parliamentary committees relating to procedural safeguards (eg, advocating against introducing judicial pre-authorisation requirements)	Impacting MAiD law reform
***Provision***		
Location of provision	**Complaints and feedback**: Letter writing advocating for no forced transfer from facility	Influencing the operation of MAiD in practice
	**Organisational involvement**: Joining as shareholder to influence institutional position	Influencing the operation of MAiD in practice
***Grief and bereavement***		
Support during and after process	**Taking on a system role**: Volunteering in peer support roles	Influencing the operation of MAiD in practice, including by: Improving accessibility (through resources) Normalising and integrating it into end-of-life care
	**Resource development**: Creating checklist for people going through process	Influencing the operation of MAiD in practice, including by: Improving accessibility (through resources) Normalising and integrating it into end-of-life care
Grief-specific resources	**Resource development**: Writing books about MAiD and grief	Influencing the operation of MAiD in practice, including by: Improving resources Normalising and integrating it into end-of-life care
***Overarching aspects targeted***		
Patient- and family-centred MAiD	**Storytelling**: Giving testimonials to bring a human face to MAiD	Influencing the operation of MAiD in practice, including by: Improving resources Normalising and integrating it into end-of-life care
	**Complaints and feedback**: Creating an online forum to increase impact of feedback	Influencing the operation of MAiD in practice
	**Representative and consultancy work**: Contributing to research	Influencing the operation of MAiD in practice, including by: Having input into decision-making about MAiD
	**Representative and consultancy work**: Representative/consultancy roles with CAMAP, DWDC, governments (local, national, international), research grants or academic conferences	Influencing the operation of MAiD in practice, including by: Improving accessibility (through resources) Having input into decision-making about MAiD
	**Resource development**: Developing resources (eg, a checklist for the MAiD process and what to expect)	Influencing the operation of MAiD in practice, including by: Improving accessibility (through resources)
	**Taking on a system role**: Incorporating lived experience into professional practice (eg, as a MAiD provider, end-of-life doula)	Influencing the operation of MAiD in practice, including by: Impacting health professionals’ practices
	**Law reform efforts**: Contributing to law-reform (eg, consulting with governments, letter-writing and brief-writing)	Impacting MAiD law reform
Creating more willing and/or better trained providers	**Storytelling**: Attending ‘before-rounds’ session to educate health professionals	Influencing the operation of MAiD in practice, including by: Increasing knowledge and awareness of MAiD amongst health professionals Impacting health professionals’ practices
	**Taking on a system role**: Taking on roles in MAiD system (eg, assessor/provider)	Influencing the operation of MAiD in practice, including by: Improving accessibility of MAiD
	**Storytelling**: Meeting with dean of university medical school to advocate for implementation of MAiD into curriculum	Influencing the operation of MAiD in practice, including by: Increasing knowledge and awareness of MAiD amongst health professionals Normalising and integrating MAiD into end-of-life care
	**Resource development**: Advising on clinical curriculum development	Influencing the operation of MAiD in practice, including by: Increasing knowledge and awareness of MAiD amongst health professionals Impacting health professionals’ practices
	**Resource development**: Reviewing resources	Influencing the operation of MAiD in practice, including by: Improving accessibility (through resources)

### B. Caregivers and patients can be regulatory actors

Individuals have been influencing MAiD law reform in Canada for some time. Caregivers and patients have impacted law reform through litigation and advocacy which resulted in MAiD being legalised,^77^ and clarified by courts,^78^ and further *Criminal Code* amendments through Bill C-7.^79^ Advocacy directed at law reform which successfully changed the final consent requirement, and successful advocacy to prevent a judicial pre-authorisation requirement being included in Bill C-14,^80^ were also reported in this study.

Our results demonstrate caregivers and patients have altered how Canadian MAiD systems operate in practice, beyond law reform, too. This includes impacting practice by taking action to fill gaps in regulation or to address system issues. Our findings are consistent with studies suggesting Canadian caregivers and patients are active participants in MAiD decision-making and care,^81^ and allies in quality improvement,^82^ and medical education.^83^ However, our findings are novel in that they map the broad range of inventive and creative actions to alter behaviour, and the significant outcomes of those actions. Our findings also highlight that caregivers and patients are part of broader ‘networks’^84^ involving advocacy organisations, religious leaders, key academics, members of parliament, and each other, seeking to alter the MAiD system.

Applying Black’s definition of sustained and focused action (element 1), directed towards altering others’ behaviour (element 2), with an intention to produce a particular outcome (element 3), some caregivers and patients are regulatory actors in Canada’s MAiD system. A clear example is that one participant, among other things, wrote a book, created a community group to share feedback, undertook representative or consultancy roles with organisations and governments, and attended academic research conferences. This variety of actions over time indicates ‘sustained and focused action’ (element 1), directed at altering behaviour of others, including by improving their practice through feedback or input into decision-making (element 2). The participant expressed motivations such as increasing patient and family voices in MAiD practice, helping others, and improving the system, demonstrating an intention to produce particular outcomes (thus also meeting the third element of Black’s definition). The actions of this participant which sought to influence the operation of MAiD in practice are regulatory, irrespective of their outcome, as outcome is not an element of Black’s definition.

Another clear example is a participant who wrote children’s books about grief and MAiD, who has also been involved in advising on a MAiD clinical curriculum (ie, sustained and focused action to alter behaviour, elements 1 and 2) with a motivation to help others and improve the system by altering behaviour (element 3). These actions were directed at influencing the operation of MAiD in practice. Finally, one participant spoke of tenacious, coordinated, and intentional advocacy efforts over a period of time (sustained and focused action, element 1) to change the final consent requirement (directed at altering behaviour and intended to change the law, elements 2 and 3), resulting in an exception to that requirement being introduced by Bill C-7. These actions were directed at influencing law reform.

These are just three examples from the data which clearly demonstrate that some caregivers and patients behave as, and can be construed as, regulatory actors by taking action to influence law reform and/or the operation of MAiD in practice. However, not all caregivers and patients in this study, or generally, are regulatory actors. For example, a person who tells the story of their experience to process their grief, and who takes no further action, may not be a regulatory actor as this action is not sustained or focused action directed at altering behaviour (though we note that there are different definitions of regulation,^85^ and if a different definition of regulation were adopted this may be considered regulatory). A person’s status as a regulatory actor is not static and may change over time. For example, a person who undertakes sustained, focused, and intentional action and then achieves their goal may step back and cease to act in regulatory ways. While not all caregivers and patients are regulatory actors, our findings suggest some caregivers and patients are capable of being construed as regulatory actors, and therefore caregivers and patients should be included in discussions of who and what impacts MAiD regulation (both in terms of law reform and the operation of MAiD in practice) in Canada.

Regulatory actions reported in this study were focused on supporting access to MAiD, but regulatory actors may also seek to advance other outcomes. This study sought to explore the experiences of persons who had accessed MAiD (primarily via reports of family caregivers) or were in the process of accessing MAiD. Our participants were all supportive of MAiD in principle and although there was diversity in experiences, family caregivers all supported their family member’s decision (as discussed further in the strengths and limitations section). Yet, some reports suggest family members who are ambivalent or opposed to MAiD or who do not support their family member’s decision may also take action to alter law reform or the operation of MAiD in practice in ways that may seek to limit access or add further safeguards.^86^ While these experiences did not arise in our sample, our findings could be applied more broadly to understand what actions (eg, storytelling) those opposed to MAiD may take to shape the MAiD system (in terms of both law reform and the operation of MAiD in practice). They may also help to understand why actions may not achieve their intended outcomes (eg, a lack of connection to advocacy groups or high-profile individuals).

### C. Patient and family caregiver regulatory action improves MAiD quality in Canada

Patients’ and caregivers’ regulatory actions impacted the functioning and operation of MAiD in practice in ways they perceived improved the system. Their actions targeted aspects of MAiD systems identified in Canadian literature that participants perceived as barriers to quality MAiD care (see also Table 4).^87^ This suggests caregivers and patients have stepped up to fill system gaps or problems in the functioning of MAiD regulation, as well as illuminating aspects of MAiD that were a problem so that these gaps and problems could be addressed by others. Through their sustained, focused, and intentional actions (ie, regulatory actions, according to Black), caregivers and patients have also contributed to what Pesut and others describe as the ‘cultural calibration’ of MAiD (ie, the calibration of systems to meet the needs of caregivers and patients, with participants in the MAiD system adjusting themselves to those demands).^88^ Participants and patients in our study actively altered the system to meet their needs, and those of other caregivers and patients, as part of this ‘cultural calibration’. We highlight three examples of barriers identified in Canadian literature,^89^ and how actions of participants sought to address these barriers to improve MAiD care and ‘calibrate’ the system.

The first barrier is lack of awareness of MAiD and process clarity.^90^ Most participants took actions (eg, storytelling) to spread awareness and educate others about MAiD and destigmatise and normalise it as an end-of-life choice; actions which may contribute to influencing how MAiD is operating in practice. This occurred within personal networks, workplaces, and more widely via advocacy organisations or media platforms. Participants perceived these actions increased MAiD awareness amongst health professionals and wider communities. Several participants also helped to develop (or improve the clarity of) MAiD resources, including through representative and consultancy work.

A second barrier is unduly burdensome legal requirements (eg, independent witnessing requirements,^91^ and the final consent requirement for all MAiD cases).^92^ Many participants became volunteer witnesses, motivated by a desire to reduce burdens on individuals seeking MAiD (and their families) to locate witnesses. Other participants (successfully) advocated to change the final consent requirement, resulting in an exception to the requirement being available in some cases (Table 1). Action by caregivers and patients to overcome what they perceived as unduly burdensome legal requirements was directed both at changing the law and at the operation of MAiD in practice (by trying to reduce the impacts of those legal requirements in the everyday practice of MAiD).

A third barrier is the impact of objections by clinicians and institutions to MAiD on patients’ and caregivers’ experiences.^93^ One participant helped develop policy guidelines about referral requirements for objecting clinicians. Another, along with the patient and BCCLA, wrote letters to a long-term care facility to persuade them (unsuccessfully) to change their position on forcibly transferring the patient away for MAiD provision. Both are examples of attempts to influence the operation of MAiD in practice. Close and others’ paper on institutional objection found patient and caregiver advocacy, particularly when amplified by the media, was a catalyst for change in institutional approaches towards MAiD and sometimes contributed to institutions allowing assessments onsite.^94^ Wiebe, in an article by Gerson and others, has also commented that patients have led change in shifting attitudes of palliative care providers towards acceptance and integration of MAiD.^95^ However, in both Australia and Canada, empirical studies found top-down regulatory responses may be needed to address institutional objections, particularly in faith-based settings.^96^ This resonates with our findings: some facilities may not respond to actions by caregivers and patients or respond to a limited extent, meaning other kinds of regulatory responses (eg, law reform efforts directed at introducing legislation or litigation) are necessary.

### D. MAiD system should be responsive to action to improve quality of MAiD regulation

Using a broad, polycentric conceptualisation of ‘regulation’ to understand the ways in which caregivers and patients impact law reform and the operation of MAiD in practice in Canada is important. It provides language to describe and identify the phenomenon, and helps to see how others are shaping the system beyond government and formal regulators. Black’s definition of regulation applied in this study requires sustained, intentional, and focused action to alter behaviour to achieve a particular outcome. This means that some caregivers and patients are going beyond a once-off action and taking repeated action directed at changing MAiD in Canada.

‘Regulatory action’ as we have defined it may be difficult for caregivers and patients to undertake,^97^ and it should not be expected of them. Some individuals may prefer not to participate in this way, a consideration which is sometimes overlooked in literature advocating for greater patient and caregiver involvement in decision-making.^98^ Additionally, they may lack power to act in regulatory ways because of systemic disadvantage, isolation, or other vulnerabilities, which are compounded by existing inherent power imbalances between caregivers and patients and other regulatory actors, like health professionals,^99^ or health institutions. This is particularly pertinent in the MAiD context, where patients, by definition, have a grievous and irremediable medical condition causing intolerable suffering. Regulatory action may be particularly burdensome when resulting from a negative experience, such as encountering a barrier to MAiD access. As key motivations of participants were helping others, filling system gaps, and changing law and policy, this suggests there are deficiencies in MAiD regulation which are (at least partially) falling to caregivers and patients to be identified, remedied or overcome (or where this is not possible, the patient may miss out on their choice of MAiD). One participant in this study explicitly raised that sometimes actions are motivated by having a traumatic experience. This resonates with research in other contexts, which has found that a key reason why individuals who experience adverse events in healthcare make complaints is to prevent other individuals from experiencing the same kind of adverse event or highlight systemic problems.^100^ It also aligns with literature that suggests that family members can have a broad range of experiences and feelings about their involvement in MAiD.^101^ For example, Crumley and others’ recent study describes some participants who experienced the ‘nuances of guilt from participating and trauma from witnessing a relative’s death, especially if it was accelerated’.^102^

Notwithstanding this, there are significant benefits to involving caregivers and patients in regulation, including improving laws (law reform) and healthcare systems (operation in practice).^103^ Caregivers and patients should be supported to undertake regulatory action *if* they wish to.^104^ For many caregivers and patients in this study, regulatory action was a meaningful, fulfilling, and rewarding undertaking. Akin to health professionals who describe MAiD work as rewarding and satisfying,^105^ many caregivers and patients reported that their role in influencing MAiD regulation afforded them a sense of purpose. When caregivers and patients are acting in regulatory ways, particularly when this action is meaningful to them, this action should be supported. A well-functioning regulatory system is responsive to patients’ and families’ concerns and actions.^106^ Regulatory systems should listen to and, where appropriate, act to investigate and address concerns raised by patients and families (including concerns about the appropriate balance between safeguards and access), who have expertise as the users of MAiD systems. As Braithwaite aptly states:

As new players come on to the field and create opportunities to build new strengths and be tripped up by new problems, smart regulators assemble the particular set of players capable of grasping contextually attuned strategies to the emerging problems and opportunities.^107^

In the MAiD context, caregivers and patients have demonstrated they should be part of the ‘assembled set’ of capable regulatory players,^108^ where they choose to be.

### E. Strengths and limitations

This is the first known study to use a regulatory lens to examine how caregivers and patients behave as regulatory actors and participate in Canadian MAiD regulation.

We collected data from caregivers across three diverse provinces, with experiences spanning six years of MAiD in Canada. This study primarily reports on caregivers’ perspectives and their accounts of patients’ experiences; we were only able to recruit one patient, as this is a difficult cohort to recruit. A limitation of the research is that caregivers’ perceptions may differ from the patient they were supporting. Their perceptions may be affected by factors such as grief and bereavement.^109^ However, evidence suggests caregivers can be reliable third-party reporters, particularly for considering the quality of end-of-life care and observable symptoms.^110^ Further research directly with patients, or with persons who are not the patient’s primary support person, may provide additional or different insights. Additionally, as existing research on the use of third-party reporters has not examined MAiD, further research on the use of third-party reporters’ accounts in this context is needed.

This study is also primarily focused on the experiences of persons with a reasonably foreseeable natural death who accessed MAiD in British Columbia, Ontario, and Nova Scotia; only one participant had a Track 2 experience. Further research with persons whose natural death is not reasonably foreseeable, as well as research with diverse experiences (eg, ineligible patients, those who missed out, diverse demographic backgrounds, and in other provinces/territories) is needed.

Our participants all supported MAiD in principle and all family caregiver participants supported their family member’s decision to access MAiD, though there was diversity in experiences. Additionally, since one form of recruitment was having the study advertisement distributed by advocacy organisations that support MAiD, some participants had direct connections to these groups. Further research with individuals who are unsupportive or ambivalent about MAiD, and with those who did not support their family member’s choice to pursue MAiD, would provide insight into their views and how they influence regulation. These individuals may also be characterised as regulatory actors and research with such individuals may result in different findings about actions, motivations, outcomes, and mediating factors.

Given our qualitative research design, our findings are not intended to be generalisable.

## V. CONCLUSION

This article challenges the framing of caregivers and patients as solely being objects of regulation in Canada’s MAiD system. Caregivers and patients should be recognised as actors with voice and agency who alter regulation, including influencing law reform and operation of MAiD in practice in Canada. For many caregivers and patients, action was meaningful and rewarding, which is positive and should be supported. Canadian MAiD regulators (and those in international jurisdictions) should be responsive to their experiences, perspectives, and their actions (regulatory or otherwise). However, the burden of taking regulatory action to address barriers to access or to quality MAiD care, or MAiD system gaps, should not fall to caregivers and patients, particularly those who do not want to regulate (or who cannot, and therefore, miss out on their choice of MAiD). Where regulatory action by caregivers and patients is necessitated by experiences of barriers to access or to quality care, or MAiD system gaps, this should signal to other regulators that consideration should be given as to how those barriers or gaps could be resolved by other regulatory means. Jurisdictions that are early in the implementation of MAiD should consider how to involve caregivers and patients to refine and improve regulation where they wish to be involved. So too should jurisdictions consider implementing MAiD in future: patient and caregiver involvement would be beneficial from the outset to ensure that MAiD practice is patient- and family/caregiver-centred.

## FUNDING

This research was funded by the Australian Research Council Future Fellowship (project number FT190100410: Enhancing End-of-Life Decision-Making: Optimal Regulation of Voluntary Assisted Dying), funded by the Australian government. The funder did not participate in or influence this research.

*Conflict of interest statement*. B.P.W. and L.W. were engaged by three Australian state governments (Victoria, Western Australia, and Queensland) to develop the legislatively mandated training for providers of assisted dying in those states. R.J. and E.C. were employed on these projects. L.W. is a member of the Voluntary Assisted Dying Review Board, the oversight body in Queensland, Australia. B.P.W. is a member of the Queensland Civil and Administrative tribunal, which has jurisdiction over some matters related to assisted dying. J.D. was involved in initiatives contributing to MAiD law reform in Canada, including the Royal Society of Canada Expert Panel: End-of-Life Decision Making; the plaintiff’s pro bono legal team in *Carter v Canada (Attorney General)* [2015] 1 SCR 331; the Provincial-Territorial Expert Advisory Group on Physician-Assisted Dying; and the Council of Canadian Academies Expert Panel on Medical Assistance in Dying. JD was also involved in developing a module for the federal MAiD National Curriculum (funded by Health Canada, developed by the Canadian Association of MAiD Assessors and Providers), and was a member of the MAID Practice Standard Task Group. JD is also on the Advisory Board for the Completed Life Initiative in the USA. All views expressed in this article are those of the authors and not the organisations they are affiliated with.

## Supplementary Material

fwae012_Supplementary_Data

## Data Availability

The data in this study are not publicly available due to confidentiality undertakings given to research participants as required by the study’s ethics approval. Requests to discuss this should be directed to the corresponding author.

## Footnotes

1 We use the terms ‘patient’ and ‘caregiver’. We acknowledge the complexities associated with ‘patient’, including it arguably implies passivity, contrary to the role we argue patients play, and may be reductive about their role as patients: see eg, Judith Healy, ‘Patients as Regulatory Actors in Their Own Health Care’ in Peter Drahos (ed), *Regulatory Theory* (1st edn, ANU Press 2017). However, we use ‘patient’ for clarity, to distinguish between caregivers and patients. We use ‘caregiver’ to refer to blood-relations and close friends.

2 The SCC allowed the Federal Government 12 months (to February 2016) to introduce legislation: see *Carter v Canada (Attorney General)* [2015] 1 SCR 331, para 147. The Federal Government requested an extension, which was permitted to June 2016. Between February and June 2016, MAiD was legal with judicial pre-authorisation, see Jocelyn Downie, ‘From Prohibition to Permission: The Winding Road of Medical Assistance in Dying in Canada’ (2022) 34 HEC Forum 321. We also focus only on the Federal MAiD law in this article. Quebec has its own distinct legislative history, see Jocelyn Downie and Kate Scallion, ‘The Path from *Rodriguez* to Bill C-14 and Beyond: Lessons about MAiD Law Reform from Canada’ in Ben P White and Lindy Willmott (eds), *International Perspectives on End-of-Life Law Reform* (1st edn, CUP 2021). However, this is outside the scope of this article.

3
*Criminal Code*, RSC, 1985, c C-46.

4 Bill C-14, *An Act to Amend the Criminal Code and to Make Related Amendments to Other Acts (Medical Assistance in Dying*), SC 2016, c 3.

5 Health Canada, ‘Fourth Annual Report on Medical Assistance in Dying in Canada 2022’ (Annual Report, 2023) <https://www.canada.ca/en/health-canada/services/publications/health-system-services/annual-report-medical-assistance-dying-2022.html> accessed 15 March 2024.

6 Joachim Cohen and Kenneth Chambaere, ‘Increased Legalisation of Medical Assistance in Dying: Relationship to Palliative Care’ (2022) 13 BMJ Supportive & Palliative Care 178.

7
*Carter v Canada (Attorney General)* [2015] 1 SCR 331 (n 2).

8 See eg, Claudia Gamondi and others, ‘Family Members’ Experiences of Assisted Dying: A Systematic Literature Review with Thematic Synthesis’ (2019) 33 Palliative Medicine 1091; Brigette M Hales and others, ‘Improving the Medical Assistance in Dying (MAID) Process: A Qualitative Study of Family Caregiver Perspectives’ (2019) 17 Palliative and Supportive Care 590; Janine Brown and others, ‘Medical Assistance in Dying: Patients’, Families’, and Health Care Providers’ Perspectives on Access and Care Delivery’ (2020) 23 Journal of Palliative Medicine 1468; Janine Brown and others, ‘Care Considerations in a Patient- and Family-Centered Medical Assistance in Dying Program’ (2020) 37 Journal of Palliative Care 341; Bernadette Roest, Margo Trappenburg and Carlo Leget, ‘The Involvement of Family in the Dutch Practice of Euthanasia and Physician Assisted Suicide: A Systematic Mixed Studies Review’ (2019) 20 BMC Medical Ethics 23; Tharshika Thangarasa and others, ‘A Race to the End: Family Caregivers’ Experience of Medical Assistance in Dying (MAiD)—A Qualitative Study’ (2022) 37 Journal of General Internal Medicine 809; Simon JW Oczkowski and others, ‘How Can We Improve the Experiences of Patients and Families Who Request Medical Assistance in Dying? A Multi-Centre Qualitative Study’ (2021) 20 BMC Palliative Care 185.

9 Judith Healy, ‘Regulatory Actors: Who Governs Health Care?’ in *Improving Healthcare Safety and Quality: Reluctant Regulators* (1st edn, Routledge 2011); Julia Black, ‘Critical Reflections on Regulation’ (2002) 27 Australian Journal of Legal Philosophy 1.

10 See eg, Hales and others (n 8); Oczkowski and others (n 8); Brown and others, ‘Medical Assistance in Dying’ (n 8); Brown and others, ‘Care Considerations’ (n 8); Ellen Wiebe and others, ‘Forced and Chosen Transfers for Medical Assistance in Dying (MAiD) before and during the COVID 19 Pandemic: A Mixed Methods Study’ (2022) 46 Death Studies 2266; Sheila Holmes and others, ‘Exploring the Experience of Supporting a Loved One through a Medically Assisted Death in Canada’ (2018) 64 Canadian Family Physician e387; Rachel Goldberg and others, ‘Impact of Medical Assistance in Dying (MAiD) on Caregivers’ (2021) 11 BMJ Supportive & Palliative Care 107; Oczkowski and others (n 8).

11 Jacynthe Rivest and others, ‘Improving Palliative Care and Medical Assistance in Dying Practice in Canada: How Patients-Partners Could Contribute to Continuing Medical Education’ (2023) 4 Palliative Medicine Reports 116.

12 See eg, Healy (n 1); Alex H Krist and others, ‘Engaging Patients in Decision-Making and Behavior Change to Promote Prevention’ (2017) 37 Information Services & Use 105; Angela Coulter and Jo Ellins, ‘Effectiveness of Strategies for Informing, Educating, and Involving Patients’ (2007) 335 British Medical Journal 24; Judith Healy, ‘Regulation by Patients’ in *Improving Health Care Safety and Quality: Reluctant Regulators* (1st edn, Routledge 2011).

13 Black (n 9).

14 Black (n 9) 26. Black defines regulation as: ‘the sustained and focused attempt to alter the behaviour of others according to defined standards or purposes with the intention of producing a broadly identified outcome or outcomes, which may involve mechanisms of standard-setting, information gathering and behaviour modification’.

15 Healy (n 12); Andrea Cornwall, ‘Unpacking “Participation”: Models, Meanings and Practices’ (2008) 43 Community Development Journal 269.

16 By ‘gaps’ we mean: (i) aspects of regulation not covered by federal or provincial law (eg, institutional objection), (ii) instances where regulatory services and systems did not exist or were underdeveloped (eg, peer and grief support), or (iii) areas where other regulation existed, but resulted in an access or other barrier that needed to be filled (eg, independent witnessing).

17
*Rodriguez v Canada (Attorney General)* [1993] 3 SCR 519; Downie and Scallion (n 2).

18
*Canadian Charter of Rights and Freedoms*, Part I of the *Constitution Act*, 1982, being Schedule B to the *Canada Act 1982* (UK), 1982, c 11.

19
*Carter v Canada (Attorney General)* [2015] 1 SCR 331 (n 2).

20 Other Canadians also provided evidence to the SCC including Elayne Shapray, who had multiple sclerosis; Leslie La Forest, who had Stage IIIC anal cancer; Peter F and Ian P, who had ALS; and Pieter Z and Jonathan L, who had Huntington’s disease. Anthony Nicklinson, from the UK, who developed locked-in syndrome after a stroke also provided evidence.

21 Several factors contributed to the decision in *Carter*, including new ‘principles of fundamental justice’ (relevant to the s 7 *Charter* argument) and evidence from permitting jurisdictions demonstrating a slippery slope had not resulted, see Downie and Scallion (n 2). Another factor was the public nature of Rodriguez’ court case which brought MAiD to the fore, see Eugene Bereza, ‘The Private and Public Deaths of Sue Rodriguez’ 39 McGill Law Journal 719.

22
*Carter v Canada (Attorney General)* [2015] 1 SCR 331 (n 2); Downie (n 2).

23 Bill C-14, *An Act to Amend the Criminal Code and to Make Related Amendments to Other Acts (Medical Assistance in Dying)*, SC 2016, c 3 (n 4).

24 ibid.

25 Downie and Scallion (n 2).

26 ibid. Patients also initiated other court applications which refined interpretation of Bill C-14, see: *AB v Canada (Attorney General)* [2017] ONSC 3759 (relating to the reasonably foreseeable natural death requirement); *Canada (Attorney General) v EF* [2016] ABCA 155 (relating to the issues of whether *Carter* required a ‘terminal illness’, and whether MAiD could be accessed for people where mental illness was the sole underlying medical condition).

27
*Lamb and British Columbia Civil Liberties Association v Canada (Attorney General)* [2016] Supreme Court of British Columbia, No S-165851.

28 This case was adjourned indefinitely after the Attorney-General’s expert gave an expansive interpretation of ‘reasonably foreseeable’, meaning a person who refused death-preventive/life-sustaining treatment would meet this criterion (effectively indicating Lamb would meet the requirement), see: ibid; Downie and Scallion (n 2).

29
*Truchon v Canada (Attorney General)* [2019] QCCS 3792.

30 ibid.

31 ibid.

32 Jocelyn Downie, ‘Medical Assistance in Dying: Lessons for Australia from Canada’ (2017) 17 QUT Law Review 127; Bill C-7, *An Act to Amend the Criminal Code (Medical Assistance in Dying)*, SC 2021, c 2.

33 Downie and Scallion (n 2); Bill C-7 ibid.

34 Alexa MacLean, ‘Audrey Parker Remembered as Nova Scotia Updates Its Assisted-Dying Policy’ (*Global News*, 10 August 2022) <https://globalnews.ca/news/9051131/audrey-parker-medically-assisted-death-nova-scotia-law/> accessed 15 June 2023; Government of Canada, ‘Canada’s New Medical Assistance in Dying (MAID) Law’ (7 July 2021) <https://www.justice.gc.ca/eng/cj-jp/ad-am/bk-di.html> accessed 18 January 2023.

35
*Audrey Parker’s Last Message to Canadians* (Directed by Dying with Dignity Canada, 2019) <https://www.youtube.com/watch?v=XwRRKq29tsw> accessed 15 June 2023.

36 MacLean (n 34).

37 ‘Personal Stories Archives’ (*Dying with Dignity Canada*) <https://www.dyingwithdignity.ca/blog/category/personal-story/> accessed 23 May 2023.

38 Special Joint Committee on Medical Assistance in Dying, ‘MEDICAL ASSISTANCE IN DYING IN CANADA: CHOICES FOR CANADIANS’ (44th Parliament, 1st Session 2023).

39 There has also been further litigation in some provinces, including a Nova Scotia case involving a wife seeking to prevent her husband, who had end-stage chronic obstructive pulmonary disease, from accessing MAiD (demonstrating patient and caregiver regulatory action can go both ways, to support and also to oppose MAiD), see eg, *Sorenson v Swinemar* [2020] NSCA 62.

40 The British Columbia Civil Liberties Association, physicians, legal experts, and advocacy organisations were also critical in this litigation.

41 David Levi-Faur, ‘Regulation and Regulatory Governance’ in David Levi-Faur (ed), *Handbook on the Politics of Regulation* (Edward Elgar 2011); Bronwen Morgan and Karen Yeung, ‘Introduction’ in Bronwen Morgan and Karen Yeung (eds), *An Introduction to Law and Regulation: Text and Materials* (CUP 2007).

42 See eg, Barbara Pesut and others, ‘Medical Assistance in Dying: A Review of Canadian Nursing Regulatory Documents’ (2019) 20 Policy, Politics, & Nursing Practice 113; Healy (n 9).

43 Black (n 9); Robert Baldwin, Martin Cave and Martin Lodge, ‘Introduction: Regulation—the Field and the Developing Agenda’ in Robert Baldwin, Martin Cave and Martin Lodge (eds), *The Oxford Handbook of Regulation* (OUP 2010); Christel Koop and Martin Lodge, ‘What Is Regulation? An Interdisciplinary Concept Analysis’ (2017) 11 Regulation & Governance 95; Jacint Jordana and David Levi-Faur (eds), ‘The Politics of Regulation in the Age of Governance’ in *The Politics of Regulation: Institutions and Regulatory Reforms for the Age of Governance* (Edward Elgar 2004); Bridget M Hutter, ‘The Role of Non-State Actors in Regulation’ (Discussion Paper No 37, April 2006); Levi-Faur (n 41); Morgan and Yeung (n 41).

44 Black (n 9) 26.

45 ibid.

46 Julia Black, ‘Constructing and Contesting Legitimacy and Accountability in Polycentric Regulatory Regimes’ (2008) 2 Regulation & Governance 137; Black (n 9).

47 Healy (n 9); Black (n 9).

48 CAMAP Canada, ‘History—CAMAP’ (2022) <https://camapcanada.ca/about/history/> accessed 14 April 2023.

49 CAMAP Canada, ‘Curriculum Project Summary—CAMAP’ (2022) <https://camapcanada.ca/curriculum/project_summary/> accessed 6 July 2023.

50 Dying with Dignity Canada, ‘About Us’ (*Dying with Dignity Canada*, 2021) <https://www.dyingwithdignity.ca/about-us/> accessed 14 April 2023.

51 Ellen Wiebe, Stefanie Green and Kim Wiebe, ‘Medical Assistance in Dying (MAiD) in Canada: Practical Aspects for Healthcare Teams’ (2021) 10 Annals of Palliative Medicine 3586; Dying with Dignity Canada (n 50).

52 See eg, Madeline Li and others, ‘Medical Assistance in Dying—Implementing a Hospital-Based Program in Canada’ (2017) 376 New England Journal of Medicine 2082; Andrea Frolic and Paul Miller, ‘Implementation of Medical Assistance in Dying as Organizational Ethics Challenge: A Method of Engagement for Building Trust, Keeping Peace and Transforming Practice’ (2022) 34 HEC Forum 371.

53 Healy (n 1); Kristin Madison, ‘Patients as “Regulators”?: Patients’ Evolving Influence Over Health Care Delivery’ (2010) 31 Journal of Legal Medicine 9; Siri Wiig and others, ‘What Methods Are Used to Promote Patient and Family Involvement in Healthcare Regulation? A Multiple Case Study across Four Countries’ (2020) 20 BMC Health Services Research 616; Siri Wiig and others, ‘Next of Kin Involvement in Regulatory Investigations of Adverse Events That Caused Patient Death: A Process Evaluation (Part I—The Next of Kin’s Perspective)’ (2021) 17 Journal of Patient Safety e1713; Ruthie Jeanneret and others, ‘“Regulatory Action” by Patients and Family Caregivers to Overcome Barriers to Accessing Voluntary Assisted Dying: A Qualitative Study in Victoria, Australia’ (2024) 47 University of New South Wales Law Journal (forthcoming); Emma Richardson and others, ‘User Involvement in Regulation: A Qualitative Study of Service User Involvement in Care Quality Commission Inspections of Health and Social Care Providers in England’ (2019) 22 Health Expectations 245; Renée Bouwman and others, ‘Patients’ Perspectives on the Role of Their Complaints in the Regulatory Process’ (2016) 19 Health Expectations 483.

54 Madison ibid.

55 Healy (n 12).

56 Jeanneret and others (n 53).

57 Queensland University of Technology, ‘Project’ (*Optimal Regulation of Voluntary Assisted Dying*) <https://research.qut.edu.au/voluntary-assisted-dying-regulation/project/> accessed 12 January 2024.

58 Joseph Maxwell, *A Realist Approach for Qualitative Research* (SAGE 2012).

59 A Tong, P Sainsbury and J Craig, ‘Consolidated Criteria for Reporting Qualitative Research (COREQ): A 32-Item Checklist for Interviews and Focus Groups’ (2007) 19 International Journal for Quality in Health Care 349.

60 Eliana Close, Jocelyn Downie and Ben P White, ‘Practitioners’ Experiences with 2021 Amendments to Canada’s Medical Assistance in Dying Law: A Qualitative Analysis’ (2023) 17 Palliative Care and Social Practice 1.

61 Ilker Etikan, Sulaiman A Musa and Rukayya S Alkassim, ‘Comparison of Convenience Sampling and Purposive Sampling’ (2016) 5 American Journal of Theoretical and Applied Statistics 1.

62 ibid.

63 Patrick Biernacki and Dan Waldorf, ‘Snowball Sampling: Problems and Techniques of Chain Referral Sampling’ (1981) 10 Sociological Methods & Research 141.

64 Virginia Braun and Victoria Clarke, ‘To Saturate or Not to Saturate? Questioning Data Saturation as a Useful Concept for Thematic Analysis and Sample-Size Rationales’ (2021) 13 Qualitative Research in Sport, Exercise and Health 201; Kirsti Malterud, Volkert Dirk Siersma and Ann D Guassora, ‘Sample Size in Qualitative Interview Studies: Guided by Information Power’ (2016) 26 Qualitative Health Research 1753.

65 Simon C Kitto, Janice Chesters and Carol Grbich, ‘Quality in Qualitative Research’ (2008) 188 Medical Journal of Australia 4.

66 Virginia Braun and Victoria Clarke, *Thematic Analysis: A Practical Guide to Understanding and Doing* (SAGE 2021); Nigel King, ‘Doing Template Analysis’ in Gillian Symon and Catherine Cassell (eds), *Qualitative Organizational Research: Core Methods and Current Challenges* (SAGE 2012).

67 Jeanneret and others (n 53).

68 Braun and Clarke (n 65); King (n 65).

69 Jeanneret and others (n 53).

70 Motivations of patients are outlined in the second category of personal motivations (ie, their actions did not relate to a different patient but rather to their own experience).

71 ‘Helping others’ is included here because we interpret ‘system’ improvement broadly. For MAiD systems to operate effectively, individuals must know MAiD is legal; know it could be an option for them; and feel comfortable to request and access it or support a patient to do so. Ideally, they also have a good experience, including sufficient supports.

72 Participants reported how their actions to support patients may have assisted patients to access their choice for MAiD, and related outcomes might be improving the patient’s access or experience of MAiD. However, as noted at the outset of the Results, because this article focuses on actions related to system-wide outcomes, we do not report these patient-facing outcomes here.

73 While the outcomes described here do not impact the system more widely, the actions resulting in these outcomes were directed towards the system, so these self-facing outcomes are a by-product of action to alter the system.

74 We note that our sample was supportive of MAiD in principle and, although there was a diversity of experiences, of their family member’s decision to access MAiD (supportiveness of MAiD or their family member’s choice was not a requirement of participation in this study). This supportiveness was sometimes expressed explicitly by participants and sometimes was implicit in the participant’s comments. Actions by individuals who were unsupportive of or ambivalent about MAiD may also be regulatory, but this is outside the scope of this paper.

75 This may also be described as an intrinsic mediating factor and overlaps with the sub-theme above: participants explained their connection to advocacy groups, particularly DWDC, was a key factor prompting them to take actions or impacting outcomes. Everyone in our sample was supportive in principle of MAiD and of their family member’s decision to seek MAiD (as noted above, this was not a requirement of participation in this study), and supportiveness of MAiD and personality and attitudes towards MAiD (described above) was, in our sample, linked to a desire to join DWDC.

76 Black (n 9).

77
*Carter v Canada (Attorney General)* [2015] 1 SCR 331 (n 2); Bill C-14, *An Act to Amend the Criminal Code and to Make Related Amendments to Other Acts (Medical Assistance in Dying)*, SC 2016, c 3 (n 4).

78
*AB v Canada (Attorney General)* [2017] ONSC 3759 (n 26); *Canada (Attorney General) v EF* [2016] ABCA 155 (n 26); *Lamb and British Columbia Civil Liberties Association v Canada (Attorney General)* [2016] Supreme Court of British Columbia, No S-165851 (n 27).

79
*Lamb and British Columbia Civil Liberties Association v. Canada (Attorney General)* [2016] Supreme Court of British Columbia, No. S-165851 (n 27); *Truchon v Canada (Attorney General)* [2019] QCCS 3792 (n 29).

80 Bill C-14, *An Act to Amend the Criminal Code and to Make Related Amendments to Other Acts (Medical Assistance in Dying)*, SC 2016, c 3 (n 4).

81 See eg, Oczkowski and others (n 8).

82 Brown and others (n 8).

83 Rivest and others (n 11).

84 Healy (n 1); Healy (n 9).

85 See eg, Philip Selznick, ‘Focusing Organisational Research on Regulation’ in Roger G. Noll (ed), *Regulatory Policy and the Social Sciences* (University of California Press 1985); Black (n 9); Baldwin, Cave and Lodge (n 43); Koop and Lodge (n 43); Morgan and Yeung (n 41); Jordana and Levi-Faur (n 43); Levi-Faur (n 41); Hutter (n 43).

86 See eg, Leyland Cecco, ‘Canada’s Assisted Dying Laws in Spotlight as Expansion Paused Again’ *The Guardian* (Toronto, 25 February 2024) <https://www.theguardian.com/world/2024/feb/25/canada-assisted-dying-laws-in-spotlight-as-expansion-paused-again> accessed 26 February 2024.

87 See eg, Hales and others (n 8); Brown and others, ‘Medical Assistance in Dying’ (n 8); Brown and others, ‘Care Considerations’ (n 8); Oczkowski and others (n 8).

88 Barbara Pesut and others, ‘Navigating Medical Assistance in Dying from Bill C-14 to Bill C-7: A Qualitative Study’ (2021) 21 BMC Health Services Research 1195.

89 Hales and others (n 8); Brown and others, ‘Medical Assistance in Dying’ (n 8); Brown and others, ‘Care Considerations’ (n 8); Oczkowski and others (n 8).

90 Oczkowski and others (n 8); Brown and others, ‘Medical Assistance in Dying’ (n 8); Hales and others (n 8).

91 Oczkowski and others (n 8); Brown and others, ‘Medical Assistance in Dying’ (n 8).

92 Brown and others, ‘Medical Assistance in Dying’ (n 8); Brown and others, ‘Care Considerations’ (n 8). Bill C-7 has introduced changes to these laws (Table 1) but because our sample reports on experiences from 2016 to 2022, this is relevant in answering our research question.

93 Hales and others (n 8); Oczkowski and others (n 8); Brown and others, ‘Medical Assistance in Dying’ (n 8).

94 Eliana Close and others, ‘A Qualitative Study of Experiences of Institutional Objection to Medical Assistance in Dying in Canada: Ongoing Challenges and Catalysts for Change’ 24 BMC Medical Ethics 71.

95 Sheri M Gerson and others, ‘Should Palliative Care Teams Be Involved in Medical Assisted Dying?’ (2023) 66 Journal of Pain and Symptom Management e233.

96 Ben P White and others, ‘The Impact on Patients of Objections by Institutions to Assisted Dying: A Qualitative Study of Family Caregivers’ Perceptions’ (2023) 24 BMC Medical Ethics 22; Close and others (n 94).

97 Healy (n 1).

98 Cornwall (n 15); Yves Longtin and others, ‘Patient Participation: Current Knowledge and Applicability to Patient Safety’ (2010) 85 Mayo Clinic Proceedings 53.

99 Healy (n 1).

100 Bouwman and others (n 53).

101 See eg, Kristie Serota, Daniel Z Buchman and Michael Atkinson, ‘Mapping MAiD Discordance: A Qualitative Analysis of the Factors Complicating MAiD Bereavement in Canada’ (2023) 34 Qualitative Health Research 195.

102 Ellen T Crumley and others, ‘Canadian Family Members’ Experiences with Guilt, Judgment and Secrecy During Medical Assistance in Dying: A Qualitative Descriptive Study’ (2023) 11 Canadian Medical Association Journal Open e782.

103 Richardson and others (n 53); Wiig and others (n 53).

104 Wiig and others (n 53).

105 Jessica Shaw, Michaela Kelly and Alanna Just, ‘Providing Medical Assistance in Dying’ 64 Canadian Family Physician e394.

106 John Braithwaite, ‘Leading from Behind with Plural Regulation’ in Judith Healy and Paul Dugdale (eds), *Patient Safety First* (1st edn, Routledge 2020).

107 ibid.

108 ibid.

109 Christine J McPherson and Julia M Addington-Hall, ‘Judging the Quality of Care at the End of Life: Can Proxies Provide Reliable Information?’ (2003) 56 Social Science & Medicine 95.

110 ibid.

